# Multimodal deep learning-based drought monitoring research for winter wheat during critical growth stages

**DOI:** 10.1371/journal.pone.0300746

**Published:** 2024-05-09

**Authors:** Jianbin Yao, Yushu Wu, Jianhua Liu, Hansheng Wang

**Affiliations:** College of Information Engineering, North China University of Water Resources and Electric Power, Zhengzhou, China; University of Brescia: Universita degli Studi di Brescia, ITALY

## Abstract

Wheat is a major grain crop in China, accounting for one-fifth of the national grain production. Drought stress severely affects the normal growth and development of wheat, leading to total crop failure, reduced yields, and quality. To address the lag and limitations inherent in traditional drought monitoring methods, this paper proposes a multimodal deep learning-based drought stress monitoring S-DNet model for winter wheat during its critical growth periods. Drought stress images of winter wheat during the Rise-Jointing, Heading-Flowering and Flowering-Maturity stages were acquired to establish a dataset corresponding to soil moisture monitoring data. The DenseNet-121 model was selected as the base network to extract drought features. Combining the drought phenotypic characteristics of wheat in the field with meteorological factors and IoT technology, the study integrated the meteorological drought index SPEI, based on WSN sensors, and deep image learning data to build a multimodal deep learning-based S-DNet model for monitoring drought stress in winter wheat. The results show that, compared to the single-modal DenseNet-121 model, the multimodal S-DNet model has higher robustness and generalization capability, with an average drought recognition accuracy reaching 96.4%. This effectively achieves non-destructive, accurate, and rapid monitoring of drought stress in winter wheat.

## Introduction

Located in the arid and semi-arid regions of China, the North China Plain is the main wheat-producing area in the country and also one of the regions frequently hit by drought. China is among the countries most severely affected by meteorological disasters worldwide, with diverse types of disasters, high intensities, and frequent occurrences. Agricultural meteorological disasters lead to significant reductions in grain production each year. The annual average grain loss nationwide is 20.628 million tons, of which drought accounts for about 60% of the total loss, resulting in an average grain reduction percentage of 4.7% [1]. Statistics show that China experiences an average of 7.5 droughts annually, affecting an average crop area of 20–30 million hm2 and leading to a grain reduction of 250–300 billion hm2. This poses a significant challenge to grain production and security [2]. The impact of drought on wheat yield and quality depends on factors such as the severity, duration, timing, and location of the drought. Research indicates that the reduction in wheat yield is not only associated with the extent of drought stress but also with the growth stage at which the stress occurs [3]. In particular, during the wheat jointing, earing, and grain-filling stages, drought stress severely affects wheat growth and yield levels, decreasing both its yield and quality [4]. Hence, obtaining real-time drought monitoring information during critical wheat growth stages, accurately identifying wheat drought stress, and promptly adopting efficient irrigation measures to prevent the intensification of drought, are fundamental for ensuring wheat drought early warning and disaster mitigation, playing a vital role in enhancing grain production.

Traditional drought monitoring methods include agricultural meteorological drought monitoring, soil moisture monitoring, thermal infrared imaging technology, hyperspectral imaging, chlorophyll fluorescence technology, and manual diagnosis. Although these methods can determine crop drought, they all have certain lag or limitations [5,6]. For example, issues such as uneven distribution of ground monitoring stations, long update cycles, limited coverage range, and excessive reliance on meteorological data. For irrigated agricultural areas, agricultural meteorological drought monitoring information has its limitations. While irrigation can alter soil moisture conditions, it cannot quickly change the air humidity and temperature in meteorological monitoring systems [7]. In comparison, soil moisture monitoring is a common indirect method. Still, due to its limited coverage and accuracy, its application faces some constraints [8]. To directly monitor crop drought stress based on the affected entity, researchers use thermal infrared imaging, hyperspectral imaging, and chlorophyll fluorescence technologies to diagnose and monitor the water status of the canopy and leaves [9]. For example, Romano et al. successfully analyzed corn’s drought resistance using thermal infrared images, selecting drought-resistant corn varieties [10]. Mangus et al., with the aid of high-resolution thermal infrared images, delved into the relationship between canopy temperature and soil moisture [11]. Although thermal infrared technology provides crop drought stress information by monitoring the temperature difference in the canopy, its spatial coverage is limited, and it’s affected by environmental conditions and crop varieties [12]. Hyperspectral technology reflects crop stress status through spectral features [13], and is widely used in crop drought stress monitoring, with the drought-sensitive band typically located between 1200nm-2500nm [14]. Chlorophyll fluorescence is sensitive to the early stages of crop drought stress, but monitoring severe drought stress using chlorophyll fluorescence parameters is challenging. Currently, chlorophyll fluorescence technology is limited to studies on small plants or crops during the seedling stage [15]. To address these issues, modern approaches utilize advanced technologies such as remote sensing, meteorological models, groundwater level monitoring, and machine learning. These technologies improve the spatiotemporal resolution of monitoring, reduce latency, and enhance the accuracy and timeliness of monitoring through the analysis of multisource data.

Currently, monitoring large crops or in-field crop phenotypes remains a challenging task. However, with the continuous advancement of computer vision and image processing technologies, deep learning methods based on two-dimensional digital images have been widely used for the identification and classification of biotic and abiotic stresses in crops [16]. Deep learning is an image recognition method that combines image feature extraction and classification. Compared to traditional machine learning, it can automatically extract image features, achieving higher recognition accuracy, and more accurately and objectively identify and grade stresses. At the same time, deep learning models have been proven to be superior to previous image recognition techniques [17], with numerous studies showing their high recognition accuracy and broad application range advantages [18,19].

In precision agriculture tasks, especially in plant monitoring, a myriad of monitoring methods have generated a significant amount of data [20]. To handle these data, there are two choices: one is to build models on each modality and evaluate their performance; the other is to combine plant growth data collected from various sources [21]. Currently, many studies have been conducted aiming to achieve multimodal data fusion. One fusion approach is to establish an integrated convolutional neural network by enhancing the contextual data of plant disease diagnosis. ContextNet is used to extract contextual data, Convolutional Neural Networks (CNN) is used for visual feature extraction, and both are integrated with the fused Mutual Correction Framework (MCF) network. This algorithm has an accuracy of 97.5% on a dataset containing 50,000 crop disease samples [22]. Another method is to develop a rice disease diagnosis model using multimodal fusion. The proposed diagnostic model can extract numerical features from data collected by sensors, visual features from images, and further combine these features with a connection layer. Results indicate that the accuracy of the multimodal fusion model exceeds that of the single modality model [23]. Despite some progress in current research on drought stress phenotypes, diagnosing crop drought stress using a single phenotype feature still has its limitations. Using multi-source sensors to obtain crop phenotype information, integrating crop color, texture, morphology, and physiological feature parameters, and employing pattern recognition algorithms to non-destructively, accurately, and quickly diagnose and monitor crop drought stress, is an important future development direction.

Therefore, this paper chooses the DenseNet-121 model to extract the phenotypic features of winter wheat during key growth stages under drought stress. It integrates agricultural meteorological data obtained through Wireless Sensor Networks (WSN) with deep learning image data, constructing the winter wheat drought stress recognition S-DNet model based on multimodal deep learning.

## Materials and methods

### Data preparation

In the experiment, the setting of the drought level during the three key growth stages of wheat refers to the requirements of the "Field Investigation and Grading Technical Specifications of Winter Wheat Disaster" Part One: Winter Wheat Drought Disaster (NY/T 2283–2012) from the Agricultural Industry Standards of the People’s Republic of China [24]. The drought levels are divided into five categories: Optimum moisture (OM), Light drought (LD), Moderate drought (MD), Severe drought (SD), and Extreme drought (ED), as shown in Table 1. Due to uneven soil moisture distribution in the field and the difficulty of accurate water replenishment, soil moisture sensors were deployed using a node deployment strategy based on the greedy ant colony algorithm, with a calibrated accuracy of ±1%. Soil moisture data was obtained by setting up soil moisture monitoring equipment in the field. Through the monitoring equipment deployed, images of wheat at different drought levels (Optimum, Light, Moderate, Severe, and Extreme) were captured, establishing a drought stress image dataset corresponding to wheat and soil moisture monitoring data.

**Table 1:** Standards for drought levels of wheat at different growth stages.

Drought Level	Rise-Jointing	Heading-Flowering	Flowering-Maturity
**OM**	>65%FC	>70%FC	>70%FC
**LD**	60%≤~<65%FC	65%≤~<70%FC	65%≤~<70%FC
**MD**	55%≤~<60%FC	60%≤~<65%FC	60%≤~<65%FC
**SD**	45≤~<55%FC	55≤~<60%FC	55≤~<60%FC
**ED**	<40%FC	<45%FC	<45%FC

Note: FC (field capacity) represents the field water holding capacity.

#### Dataset description

**Fig 1:**
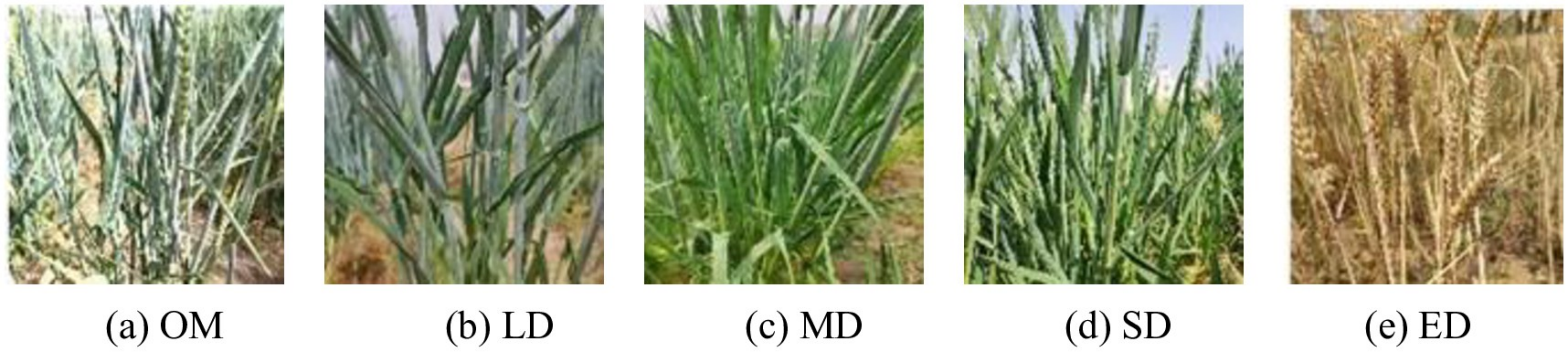
Sample of some winter wheat.

The experiment was conducted from April 2021 to June 2022 in the Efficient Agricultural Water Use Laboratory of North China University of Water Resources and Electric Power. The experiment selected three stages of winter wheat that are significantly affected by drought stress: rise-jointing (RJ), heading-flowering (HF) and flowering-maturity (FM). By monitoring soil moisture sensors in real-time, sample images of wheat at different drought levels during the three key growth stages were collected. After annotation and screening, a total of 12,500 images (see Table 2) were used for model training. The time of wheat image collection is shown in Table 3, and some samples of winter wheat images are shown in Fig 1.

**Table 2:** Number of wheat images at different growth stages.

Growth Stage	OM	LD	MD	SD	ED	Total Data
**RJ**	950	900	880	820	650	4200
**HF**	900	840	840	845	775	4200
**FM**	1220	920	850	720	490	4100

**Table 3:** Acquisition time of wheat drought images.

Year	RJ(Month/Day)	HF(Month/Day)	FM(Month/Day)
**2021**	04.03–04.23	04.24–05.02	05.03–05.23
**2022**	03.29–04.16	04.17–04.30	05.01–05.19

Sensors and mini weather stations were deployed in the field research area to collect agricultural meteorological data (as shown in Table 4). Monitoring equipment was used to obtain wheat drought stress image data. Meteorological data was collected through temperature sensors, air humidity sensors, soil moisture sensors, light sensors, pH sensors, rainfall sensors, wind speed and direction sensors, ground net radiometers, etc.; soil information was gathered through soil pH values, soil moisture, and soil heat flux, etc.

**Table 4:** Agricultural meteorological non image data samples collected through sensors.

Agricultural Meteorological Index	OM	LD	MD	SD	ED
Temperature* **(°C)**	25.4	27.5	32.8	37.0	42.2
Relative Humidity* **(%)**	62	54	45	33	25
Soil Temperature* **(°C)**	20.2	22.4	26.1	30.4	34.1
**Soil pH Value** *	6.5	6.2	5.8	5.3	4.7
**Soil Moisture** #	Moist	Moist	Moderate	Dry	Dry
**Sunlight Intensity** #	Weak	Moderate	Moderate	Strong	Strong

* indicates the average value in calculation,

^#^ indicates that the index data is multi-valued.

### Proposed framework

**Fig 2:**
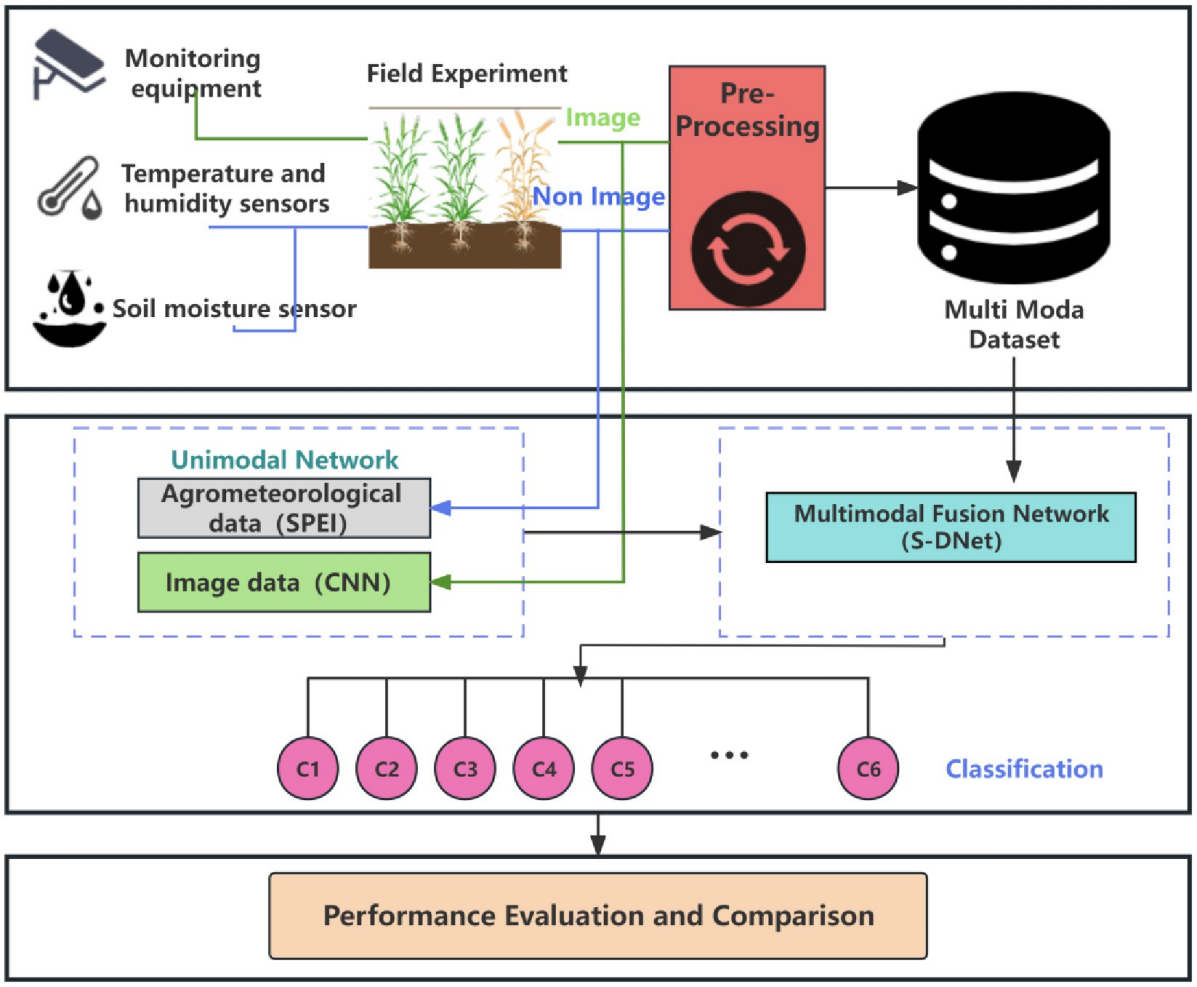
The overall workflow of S-DNet framework.

For precision agriculture tasks, fusing multiple data sources can enhance the understanding of real-world scenarios [25]. Thus, this section introduces an end-to-end multi-modal framework for winter wheat phenotypic analysis. This framework employs meteorological drought data to describe drought characteristics, combined with a deep learning model to identify winter wheat phenotypic drought traits. The overall workflow of the model is depicted in Fig 2. Compared to traditional CNN architectures, an added digital agriculture meteorological data module extracts meteorological drought traits, further enhancing perception in real data scenarios when fused with image drought traits. The next section will discuss the architecture of these baseline models and the proposed multimodal fusion technology.

#### Baseline 1: SPEI

The most widely used in the monitoring and analysis of meteorological drought are the Standardized Precipitation Index (SPI) [26] and the Palmer Drought Severity Index (PDSI) [27]. However, in the monitoring of meteorological drought, one index cannot comprehensively and objectively reflect the real situation of dry and wet surface [28].

To fully leverage the advantages of both PDSI and SPI indices, the Standardized Precipitation Evapotranspiration Index(SPEI) was developed. The SPEI was proposed by Vincente-Serrano et al [29], and is built on the SPI by introducing the potential evapotranspiration term, integrating the effects of precipitation and temperature on evapotranspiration. In some regions of China, the SPEI index has been applied to meteorological drought studies. For example: Safwan Mohammed et al. examined the intensity, duration, and severity of agricultural drought using the SPI and SPEI for Hungary from 1961 to 2010. They revealed the impact of drought on maize and wheat yields by analyzing standardized yield residuals and crop-drought elasticity factors [30]; Cheng Junqi et al. took Xinjiang as an example and analyzed the increase in drought frequency in China due to global warming based on the SPEI index from 1961 to 2020. They studied the impact on cotton, wheat, and maize yields [31]; Shengli Liu et al. focused on summer maize in the Huang-Huai-Hai agricultural region of China. They quantitatively analyzed the impact of drought on crop yields using annual phenological data and the SPEI from 1981 to 2010 [32]; Liu Ying et al. utilized various data sources, including CRU precipitation data, to study drought propagation and the impact of water resources on vegetation in the karst region of southwestern China. They employed the SPI and the random forest method for their research [33].

SPEI is built on the SPI by introducing the potential evapotranspiration term, and like the SPI, SPEI is also a drought index based on a probability model. The calculation steps of SPEI are as follows:

Step one: Use the PenMan-Monteith formula [34] revised by the United Nations Food and Agriculture Organization to calculate crop evapotranspiration. The specific calculation formula is as follows:
$$ET_{0}=\frac{0.408\Delta (R_{n}-G)+\gamma \frac{900}{T+273}u_{2}(e_{s}-e_{a})}{\Delta +\gamma (1+0.34u_{2})}$$ (1)
  Where: *ET*_0_ is the crop evapotranspiration, mm; *R*_*n*_ is the net radiation at the ground surface, MJ / (m^2^ · d^1^); *G* is the soil heat flux, MJ / (m^2^ · d^1^); *γ* is the psychrometric constant; *T* is the daily average temperature, °C; *U*_2_ is the wind speed at 2 meters height, *m*/*s*; *e*_*s*_ is the saturated vapor pressure; *e*_*a*_ is the actual vapor pressure; Δ is the slope of the vapor pressure curve.
Step 2: Calculate the difference *D*_*i*_ between daily precipitation and potential evapotranspiration:
$$D_{i}=P_{i}-PET_{i}$$ (2)
  Where: *P*_*i*_ is the precipitation, mm; *PET*_*i*_ is the potential evapotranspiration on day *i*, mm.
Step 3: Establish the moisture surplus/deficit cumulative series at different time scales:
$$D_{n}^{k}=\sum_{i=0}^{k-1}\left(P_{n-i}-PET_{n-i}\right)\text{,}n\geq k$$ (3)
  Where: *k* is the time scale (days); *n* is the total number of days.
Step 4: Normalize the $D_{n}^{k}$ data series, and the normalized value is the SPEI value. Vicente-Serrano compared the fitting effects of the Log-logistic, Pearson, Log-normal, and Generalized Extreme Value on the $D_{n}^{k}$ series. The results showed that the Log-logistic distribution has the best fitting effect on the $D_{n}^{k}$ series, and the estimation method of the fitting parameters uses the linear moment method [35].
  Using the three-parameter log-logistic probability distribution to normalize the *D*_*i*_ data series, calculate the SPEI index corresponding to each value:
$$F(x)={\left[1+{\left(\frac{\alpha }{x-\gamma }\right)}^{\beta }\right]}^{-1}$$ (4)
$$\alpha =\frac{(\omega_{0}-2\omega_{i})\beta }{\Gamma (1+1/\beta )\Gamma (1-1/\beta )}$$ (5)
$$\beta =\frac{2\omega_{1}-\omega_{0}}{6\omega_{1}-\omega_{0}-6\omega_{2}}$$ (6)
$$\gamma =\omega_{0}-\alpha \Gamma (1+1/\beta )\Gamma (1-1/\beta )$$ (7)
$$\omega_{s}=\frac{1}{N}{\sum_{i-1}^{N}\left(1-F_{i}\right)}^{s}D_{i}$$ (8)
$$F_{i}=\frac{i-0.35}{N}$$ (9)
  Where: *F*(*x*) represents the probability density function; *x* is the independent variable of the probability density function; Parameters α, β, and γ are respectively the scale, shape, and origin parameters; Γ is the factorial function; *ω*_*s*_ is the probability-weighted moment of the data series *D*_*i*_; s is the ordinal number of the probability-weighted moment, s = 0, 1, 2; *N* represents the number of times used in the calculation.
Step 5: Standardize the probability distribution function *F*(*x*) of the difference *D*_*i*_ between precipitation and evaporation. Let *P* = 1 − *F*(*x*):
  When the cumulative probability *P* ≤ 0.5, $\omega =\sqrt{-2\text{ln}(P)}$, then the formula for *SPEI* is:
$$SPEI=\omega -\frac{c_{0}+c_{1}+c_{2}\omega^{2}}{1+d_{1}\omega +d_{2}\omega^{2}+d_{3}\omega^{3}}$$ (10)

When the cumulative probability *P* > 0.5, $\omega =\sqrt{-2\text{ln}(1-P)}$, then the formula for *SPEI* is

$$SPEI=-(\omega -\frac{c_{0}+c_{1}+c_{2}\omega^{2}}{1+d_{1}\omega +d_{2}\omega^{2}+d_{3}\omega^{3}})$$ (11)

Where: *c*_0_ = 2.515 517, *c*_1_ = 0.802 853, *c*_2_ = 0.010 328, *d*_1_ = 1.432 788, *d*_2_ = 0.189 269, *d*_3_ = 0.001 308.

Based on SPEI, the drought level classification is shown in Table 5.

**Table 5:** Drought classification of SPEI.

Drought Level	OM	LD	MD	SD	ED
**SPEI Value**	(−0.5, 0.5]	(−1, −0.5]	(−1.5, −1]	(−2, −1.5]	(−∞,−2]

#### Baseline 2: DenseNet-121

**Fig 3:**
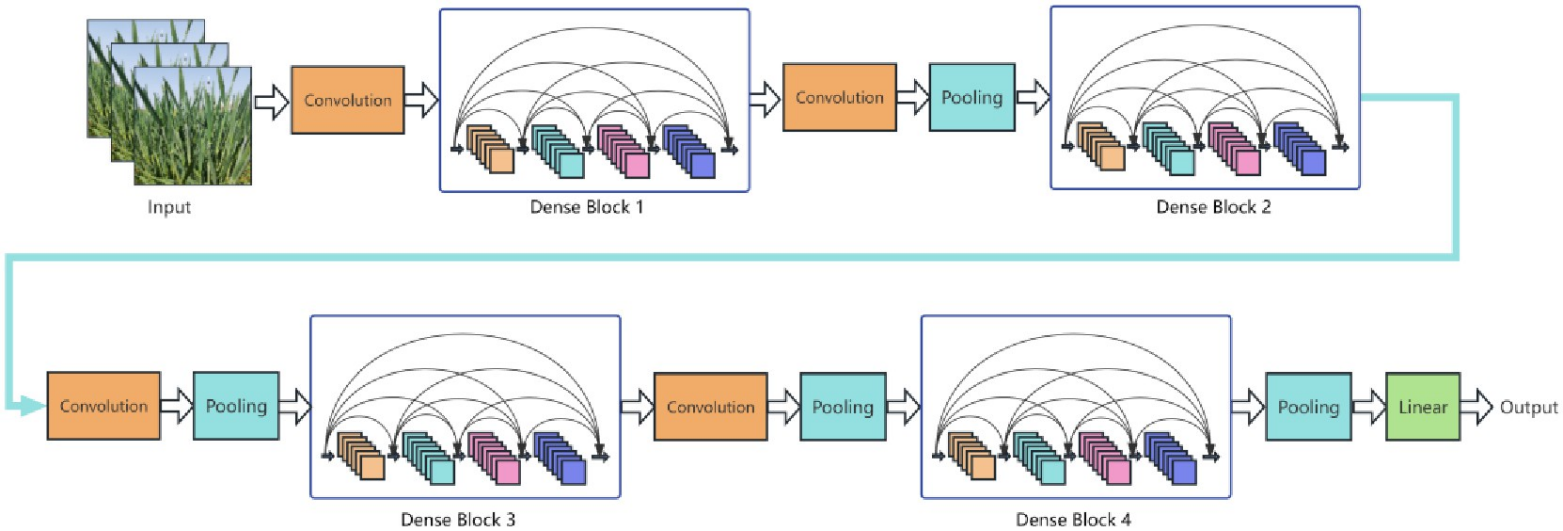
DenseNet network structure diagram.

DenseNet (Densely Connected Convolutional Network) is a deep convolutional neural network structure proposed by Gao et al. in 2019 [36]. The network structure of the DenseNet series is shown in Fig 3. Unlike traditional convolutional neural networks, the output of each layer in DenseNet is connected with the outputs of all previous layers, forming a densely connected structure. This kind of connection ensures more thorough feature propagation, effectively reducing the vanishing gradient problem, and enhancing both the training efficiency and generalization capacity of the model. DenseNet-121 consists of 121 layers. This network adopts a new architecture that is both concise and efficient, demonstrating superior performance over the Residual Network (ResNet) on the CIFAR metric.

#### Multimodal fusion

**Fig 4:**
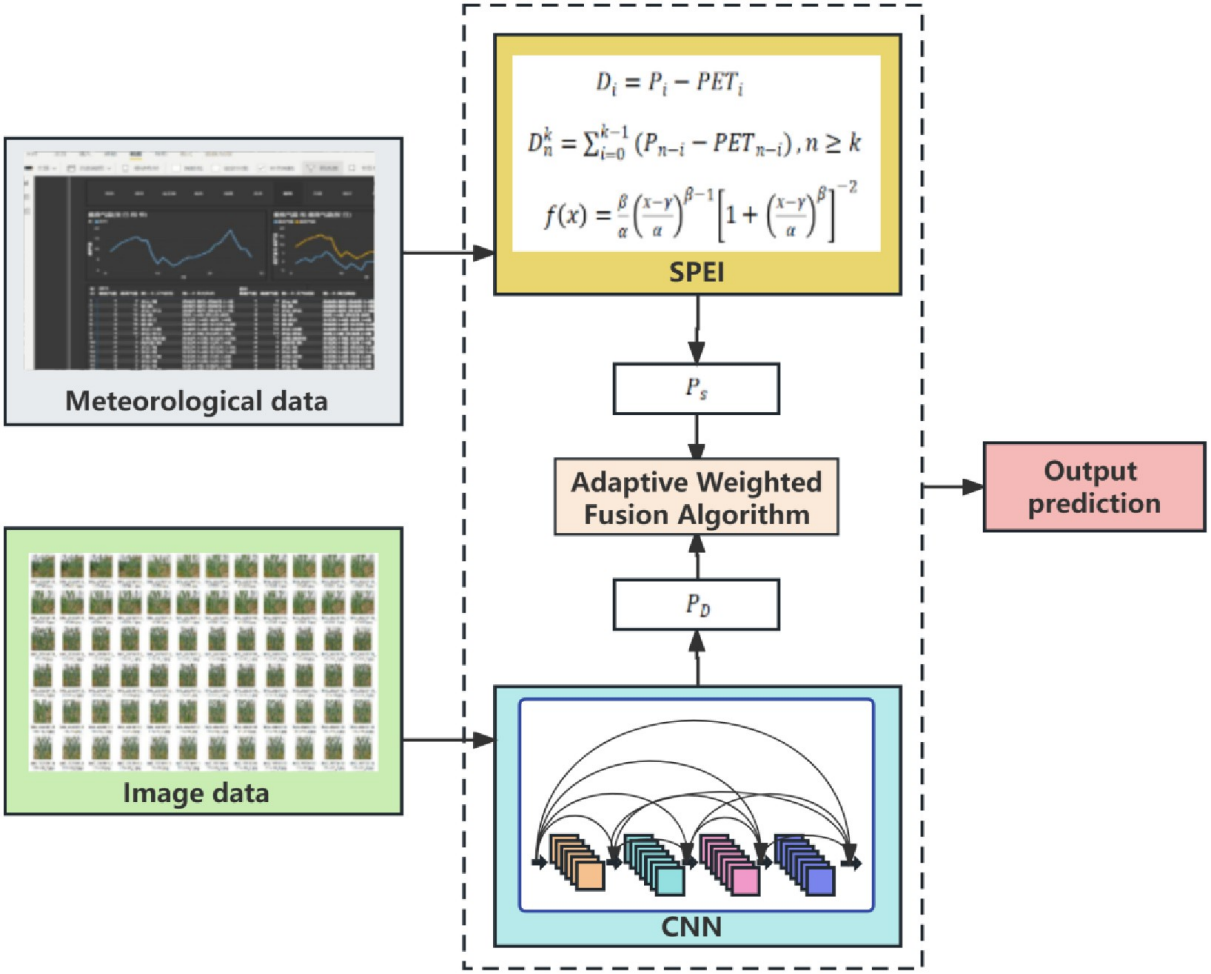
Architecture of proposed S-DNet framework.

When employing deep learning models for image classification, the prediction results are typically given as a probability distribution, with each category receiving a confidence score. However, relying solely on image classification results may not meet the needs of practical applications, especially when other relevant information is combined with image classification, the current digital agrometeorological data includes meteorological and soil-related information, such as temperature, air humidity, light intensity, wind speed, soil moisture, precipitation, trace elements, soil pH value, etc. After fusing data from different sources, the network is more elastic, fault tolerance and accuracy than when using only one data source. By merging winter wheat phenotypic image traits with SPEI text traits, we enhance model performance, resulting in a drought monitoring model called S-DNet, which integrates SPEI with DenseNet-121. The model’s framework is shown in Fig 4.

**Fig 5:**
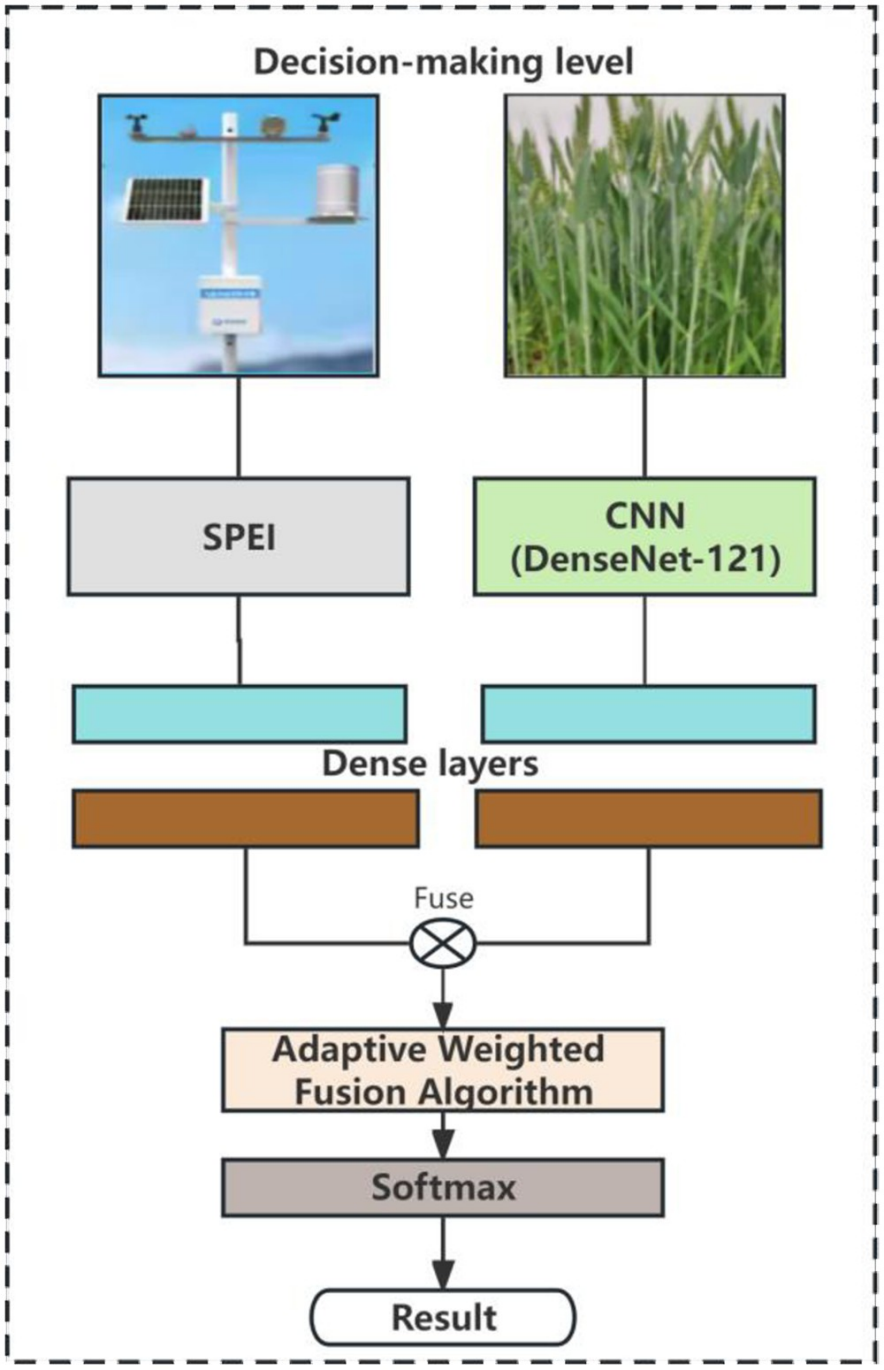
Decision layer framework.

Among them, the basic idea of decision layer fusion is to use an adaptive weighted fusion method, merging the probability vector of different meteorological drought levels derived from SPEI with the probability vector of wheat drought levels identified by the DenseNet-121 model. This method allows for the organic combination of the prediction results of both approaches, fully utilizing each of their feature information, and thus yielding a more comprehensive drought probability vector. The framework of decision layer fusion is shown in Fig 5. Before the data fusion at the decision layer, it’s imperative to ensure that the drought probability vectors from SPEI and DenseNet-121 model are consistent, ensuring both modalities output the same drought categories, laying the groundwork for consistent fusion. At the same time, depending on the real-time meteorological conditions, weights are allocated to the probability vector of each method, ensuring a balance among various factors.

#### Evaluation metrics

This research uses several metrics to evaluate the winter wheat drought stress identification and grading model, including the accuracy of drought stress identification (*A*1), the precision of drought stress classification (*F*1), and the comprehensive evaluation metric *F*1 score. The accuracy *A*1 evaluates the precise degree of drought identification, the precision *P*1 assesses classification results, and the *F*1 score is the harmonic mean of precision and recall, evaluating the model’s identification accuracy for winter wheat drought images, integrating the strengths and weaknesses of both.

1. Accuracy (*A*1) represents the proportion of samples correctly classified by the classifier to the total number of samples, calculated as:
$$A_{1}=\frac{\text{TP}+\text{TN}}{\text{TP}+\text{TN}+\text{FP}+\text{FN}}$$ (12)
2. Precision (*P*1) refers to the proportion of actual positive samples in each category predicted as positive, calculated as:
$$P1=\frac{\text{TP}}{\text{TP}+\text{FP}}$$ (13)
3. Recall (*R*1) represents the proportion of positive samples in each category predicted as positive, calculated as:
$$R_{1}=\frac{\text{TP}}{\text{TP}+\text{FN}}$$ (14)
4. The *F*1 score is a comprehensive evaluation metric, which is the harmonic mean of precision and recall. The higher the *F*1 score, the better the classifier’s performance. The formula for calculating the *F*1 score is:
$$F1=\frac{2P_{1}R_{1}}{P_{1}+R_{1}}$$ (15)

In Table 6: TP represents the number of true positive samples predicted as positive by the model; TN denotes the number of true negative samples predicted as negative by the model; FP stands for the number of actual positive samples predicted as negative; FN signifies the number of actual negative samples predicted as positive.

**Table 6:** The confusion matrix for determining whether the classification of winter wheat is correct based on the recognition model.

Label Category	Model Prediction		
		0	1
**Truth Label**	0	True positive (TP)	False negative (FN)
	1	False positive (FP)	True negative (TN)

## Results and discussion

### Multimodal fusion results

In this section, the adaptive weighted fusion method is used to merge the drought probability vectors from SPEI and DenseNet-121 model, aiming to enhance the accuracy and robustness of drought level prediction. This method allows the prediction results from both approaches to be organically combined, fully utilizing their respective information, resulting in a more comprehensive drought probability vector [37]. Before data fusion, it’s necessary to obtain the drought probability vectors of the SPEI and DenseNet-121 model and ensure that both methods output the same drought categories, thus laying the groundwork for vector fusion. The specific computation is as follows:

$$P=\omega_{1}P_{S}+\omega_{2}P_{D}$$ (16)

**Fig 6:**
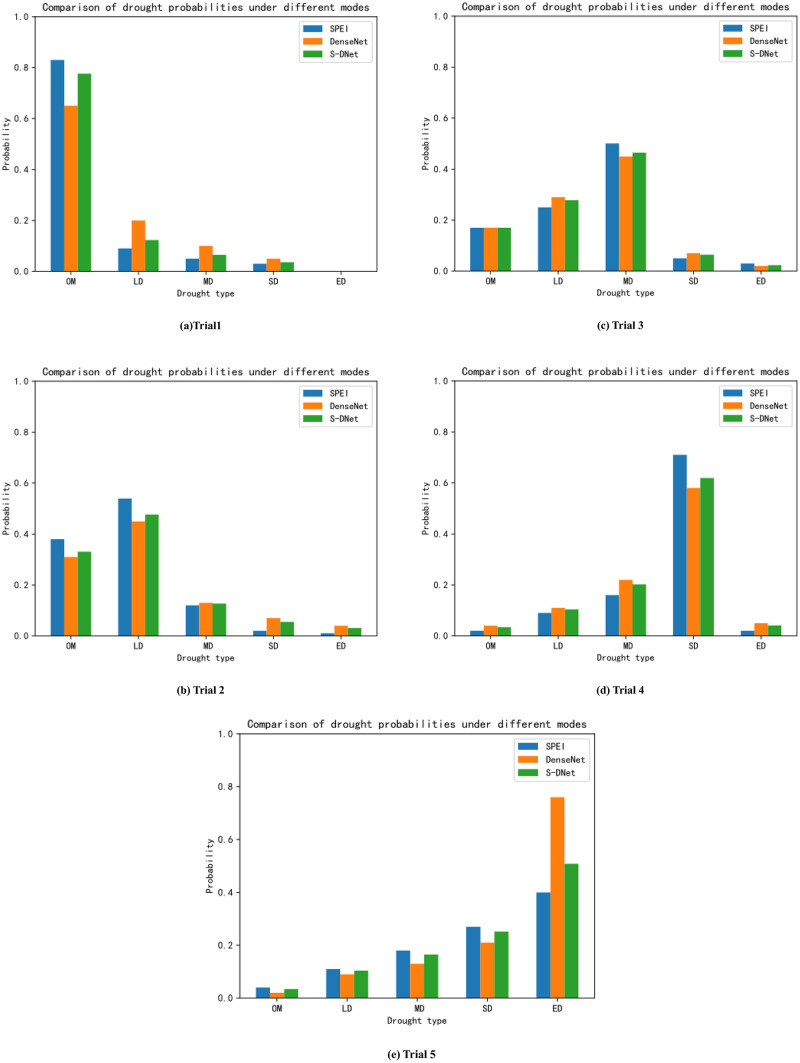
Comparison of multimodal drought probability.

Where *ω*_1_ and *ω*_2_ are the weights of the SPEI index method and DenseNet-121 method, respectively, and *ω*_1_ + *ω*_2_ = 1. Moreover, to ensure the uniformity and interpretability of the fusion results, the merged probability vector is normalized to ensure the sum of the probabilities is 1. Aligning the SPEI data with the deep learning model output data in time and space enables a comparison within the same temporal scale and geographical scope. This yields a comparison bar chart of drought probabilities under multimodal conditions, as shown in Table 7 and Fig 6. They display the drought probability vectors and comparison charts obtained from SPEI, DenseNet model, and S-DNet method under different drought levels.

**Table 7:** Drought probability vector under S-DNet method.

Trial	SPEI					DenseNet					S-DNet				
	OM	LD	MD	SD	ED	OM	LD	MD	SD	ED	OM	LD	MD	SD	ED
**1**	**.83**	.09	.05	.03	-	**.65**	.20	.10	.05	-	**.77**	.12	.07	.04	-
**2**	.38	**.54**	.12	.02	.01	.31	**.45**	.13	.07	.04	.33	**.48**	.13	.06	.03
**3**	.17	.25	**.50**	.05	.03	.17	.29	**.45**	.07	.02	.17	.28	**.47**	.06	.02
**4**	.02	.09	.16	**.71**	.02	.04	.11	.22	**.60**	.05	.03	.10	.20	**.62**	.04
**5**	.04	.11	.18	.27	**.40**	.02	.09	.13	.21	**.76**	.03	.10	.16	.25	**.50**

Fig 6 illustrates the comparison results of drought probabilities under multimodal conditions. As evident from the bar charts, by using the adaptive weighted fusion method, the S-DNet model can harness the advantages of both modalities, yielding a slight increase in the final drought prediction probability compared to single-modal image recognition, thereby enhancing the precision and reliability of drought level prediction through image data.

### Comparative analysis

**Fig 7:**
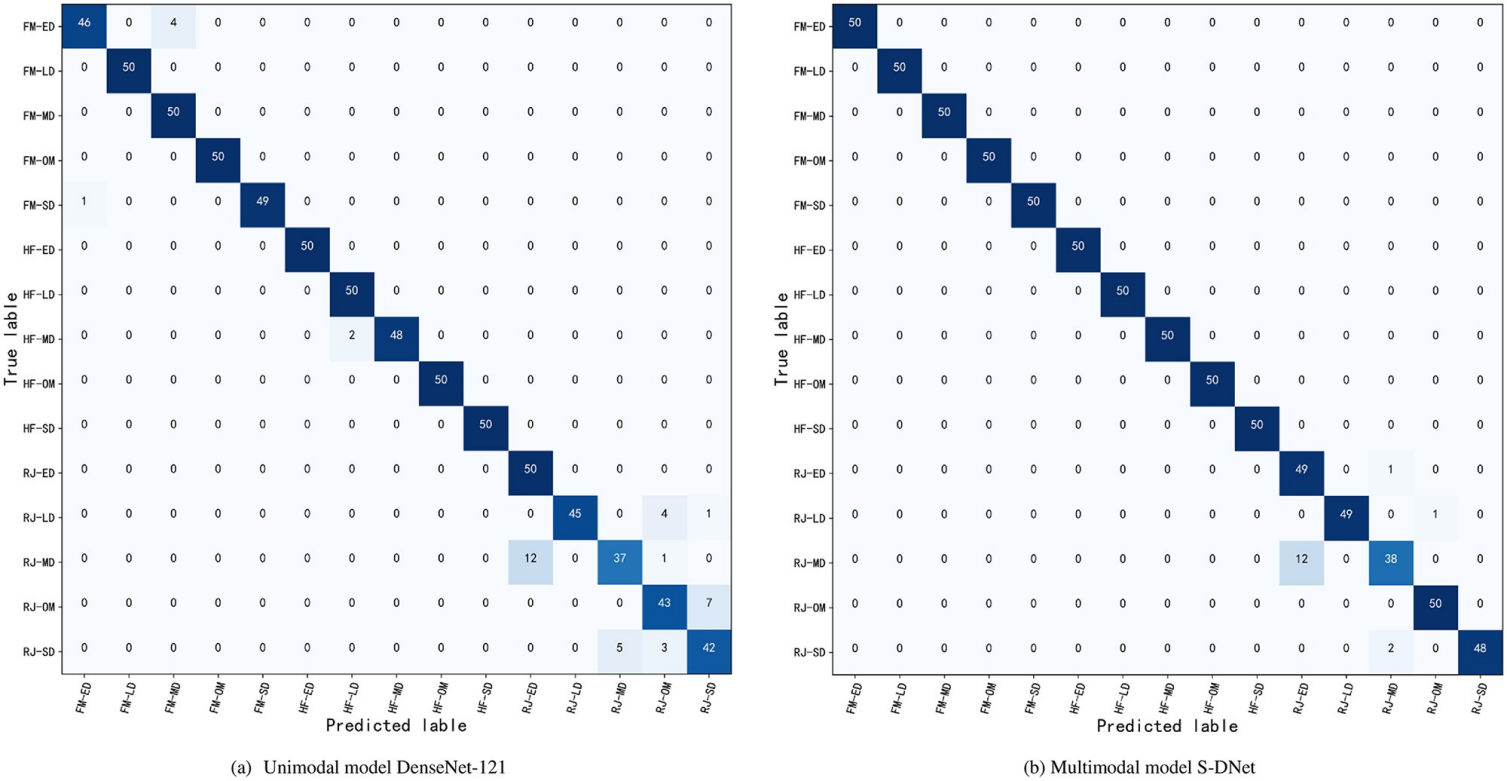
The confusion matrix of unimodal and multimodal models.

In order to further improve the accuracy of the winter wheat drought identification model, SPEI is calculated based on the agricultural meteorological data obtained from WSN and fused with the convolutional neural network (CNN) model, DenseNet-121, which was pre-trained using image data. A multi-modal fusion network framework, SPEI-DenseNet-121 (S-DNet), is proposed. By learning the features of both image and non-image data, combined with deep learning classification techniques, a study on the drought conditions during the three key growth stages of winter wheat under drought stress is carried out. A performance evaluation comparison test is conducted between the unimodal DenseNet-121 model and the multimodal S-DNet model. In the experiment, randomly initialized weights were used; both unimodal and multimodal used the same SGD optimizer for training, with a batch size of 64 and a fixed learning rate of 0.001, employing a gradual learning rate strategy. Performance evaluation indicators including classification accuracy, loss values, and F1 scores were calculated to assess the performance of the various models. The results are shown in Table 8 and Fig 7.

**Table 8:** Performance of unimodal and multimodal models.

	Mode	Data	Structure	Growth Stage	Accuracy	Loss	*F*1 Score
**1**	Unimodal (DenseNet-121)	Image	CNN	RJ	84.4%	0.221	0.8322
**2**		Image	CNN	HF	99.2%	0.097	0.9891
**3**		Image	CNN	FM	97.2%	0.120	0.9649
**4**	Multimodal (S-DNet)	Fusion	SPEI+CNN	RJ	90.0%	0.181	0.8921
**5**		Fusion	SPEI+CNN	HF	99.6%	0.081	0.9957
**6**		Fusion	SPEI+CNN	FM	99.6%	0.082	0.9956

The confusion matrix [38] is one of the tools used to evaluate model factors. It is a matrix-style heatmap where rows represent true category labels and columns represent predicted outcomes. The confusion matrices for the unimodal DenseNet-121 model and the multimodal S-DNet model results are shown in Fig 7.

To enhance the accuracy of drought degree monitoring during the key growth stages of wheat, a deep learning fusion strategy based on SPEI was explored. This involved integrating crop image data with non-image agricultural meteorological data collected by sensors, leading to the proposal of a multimodal fusion S-DNet network model. The model recognizes and classifies the drought degree of wheat during its key growth stages based on WSN data features and image learning features. Results show that: ① Compared to the unimodal DenseNet-121 network model, the S-DNet has superior accuracy, robustness, and practicality. It displayed significantly better performance when identifying and grading the drought degree during wheat’s key growth stages, with an average identification accuracy of 96.4%. ② The multimodal fusion S-DNet model’s drought identification accuracy surpassed that of the unimodal DenseNet-121 model, improving the average model identification accuracy by 2.8 percentage points across the three key stages. ③ Compared to the confusion matrix of the unimodal DenseNet-121, the multimodal fusion confusion matrix better captures the interrelationships between different modes, thereby enhancing classification accuracy and reliability. ④ By fusing deep learning’s DenseNet-121 model with SPEI meteorological data, the model is better equipped to understand and grasp the inherent patterns in the data. This multimodal fusion method offers a more comprehensive and enriched information, enhancing the model’s robustness and generalizability.

## Conclusion

In conclusion, this study proposed a novel multimodal deep learning approach for monitoring drought stress in winter wheat, aiming to improve the accuracy and efficiency of drought stress assessment during critical growth stages. By collecting and analyzing drought stress images of winter wheat at the Rise-Jointing, Heading-Flowering, and Flowering-Maturity stages, a dataset corresponding to soil moisture monitoring data was established. The DenseNet-121 model was employed as the base network to extract drought features, and a multimodal deep learning-based S-DNet model was developed by integrating meteorological factors, IoT technology, and the meteorological drought index SPEI obtained through WSN sensors.

The results demonstrate that the multimodal S-DNet model significantly outperforms the single-modal DenseNet-121 model, achieving an average drought recognition accuracy of 96.4%. This indicates the model’s high robustness and generalization capability, enabling non-destructive, accurate, and rapid monitoring of drought stress in winter wheat. The study’s findings suggest that the multimodal fusion network provides a reliable and effective approach for evaluating drought stress in winter wheat, with broad applications in agricultural production and resource management.
